# Supplementary material for: Systems serology detects functionally distinct coronavirus antibody features in children and elderly
Source: Nat Commun. 2021 Apr 1;12:2037. doi: 10.1038/s41467-021-22236-7 (PMC8016934; doi:10.1038/s41467-021-22236-7)
Supplement: Supplementary file 1 — Supplementary information. [file 41467_2021_22236_MOESM1_ESM.pdf]

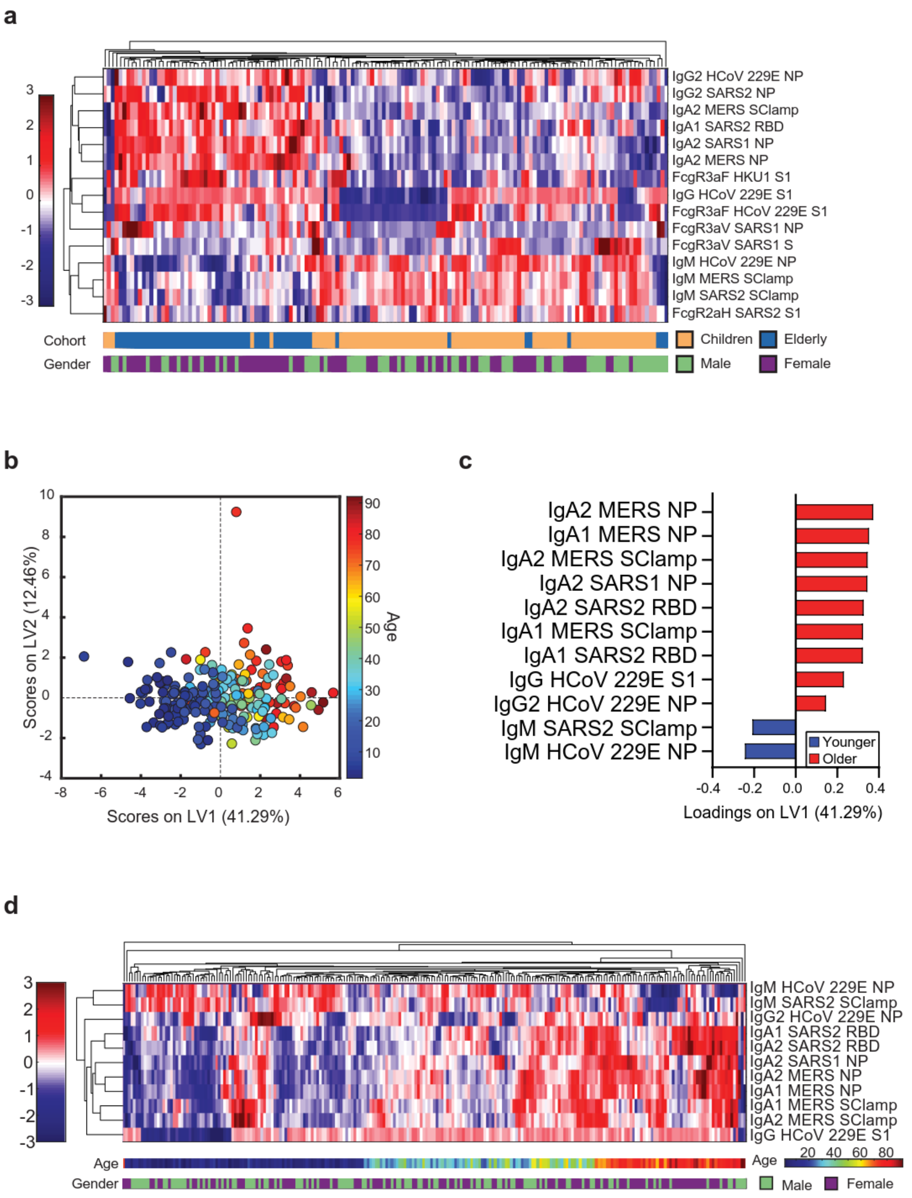

Supplementary Figure 1: SARS-CoV-2 serological signatures differ between healthy children, adults and elderly

(a) Hierarchical clustering of Elastic-Net selected features for children and elderly. Levels are coloured from low (dark blue) to high (dark red). PLSR model scores plot (b) loadings plot (c) for all healthy patient data on Elastic-Net 11-feature signature. The model goodness of fit (R2) was 0.6421 and goodness of predication (Q2) was 0.6144. Percent variance explained by each latent variable in parentheses. (d) Hierarchical clustering of signature in (b-c). Multiplex was performed in duplicate.

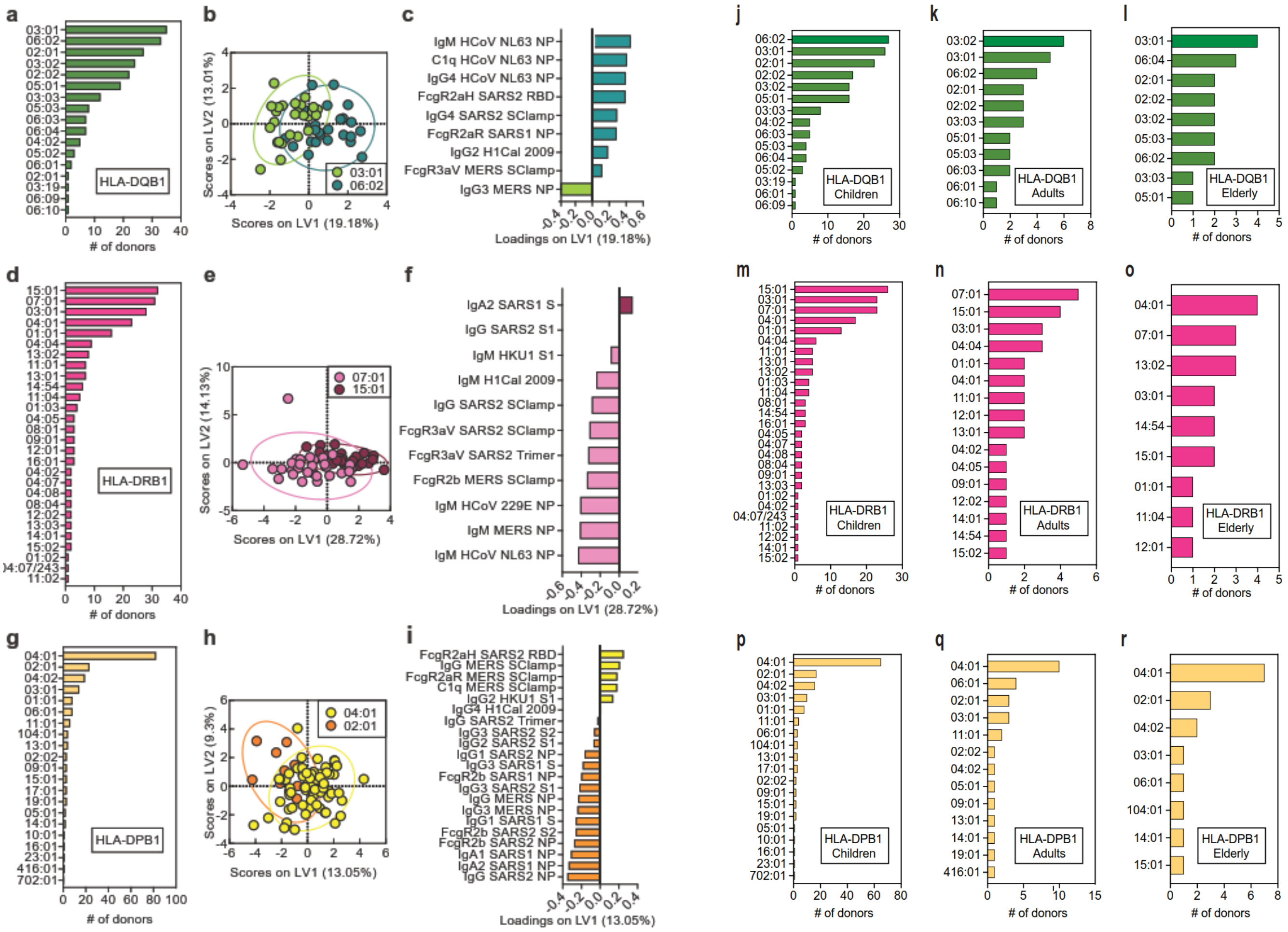

Supplementary Figure 2: HLA class II alleles influence Ab signatures

HLA-DQB1, -DRB1 and -DPB1 alleles in our healthy donor cohort (a,d,g), PLSDA scores (b,e,h) and loadings (c,f,i) plots using the Elastic-Net selected signatures for the two most frequent alleles (HLA-DQB1*03:01 and 06:02, 82.59% cross-validation accuracy, 86.44% calibration accuracy; HLA-DRB1*15:01 and 07:01, 76.32% cross-validation accuracy, 77.83% calibration accuracy; HLA-DPB1*04:01 and 02:01, 74.23% cross-validation accuracy, 82.44% calibration accuracy). Variance explained on each LV is in parentheses. Analysis was performed on a subset of the healthy individuals (n=111) for whom HLA class II type was available. Donors heterozygotes for the two most frequent HLA alleles were excluded from PLSDA and loading analysis. Distribution of HLA-DQB1 (j-l; green), -DRB1 (m-o; pink) and -DPB1 (p-f; yellow) alleles detected in healthy donor children (j, m, p), adults (k, n, q), and elderly (f, o, p). HLA class II alleles analyzed in figure 4 are indicated by a bold outline.

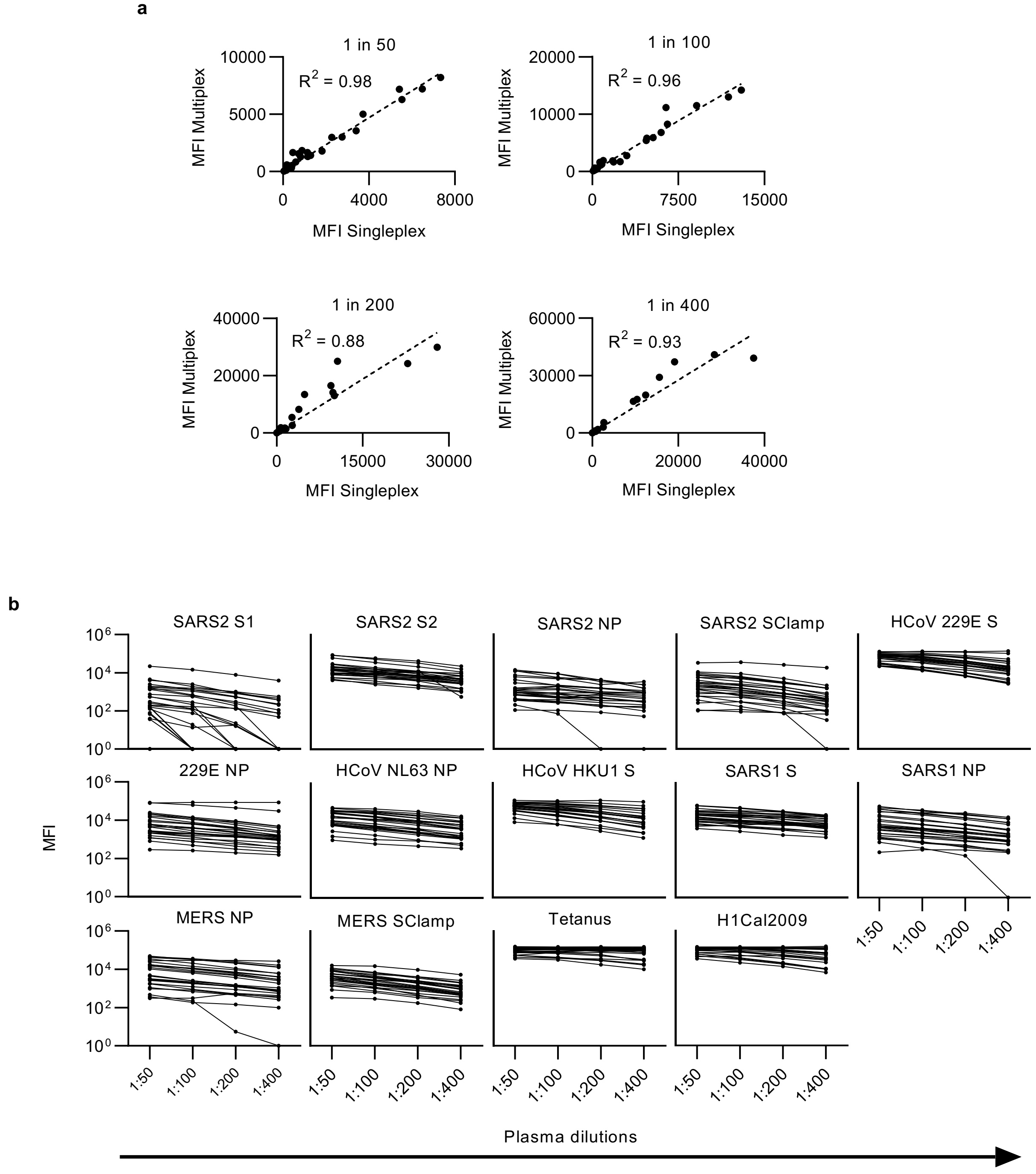

Supplementary Figure 3: Multiplex assay setup and optimization

(a) Multiplex was validated by measuring a subset of healthy samples both in singleplex and multiplex. Strong correlations suggest that multiplexing did not affect measurement of Ab responses, especially at 1:50 and 1:100 dilutions. (b) Serial dilution of a subset of healthy samples against 14 antigens included in the custom multiplex assay to select dilutions where saturation was not observed. A final dilution of 1: 100 showed not only strong correlation between singleplexed and multiplexed antigens but was also not saturated for most antigens and was selected for subsequent assays.

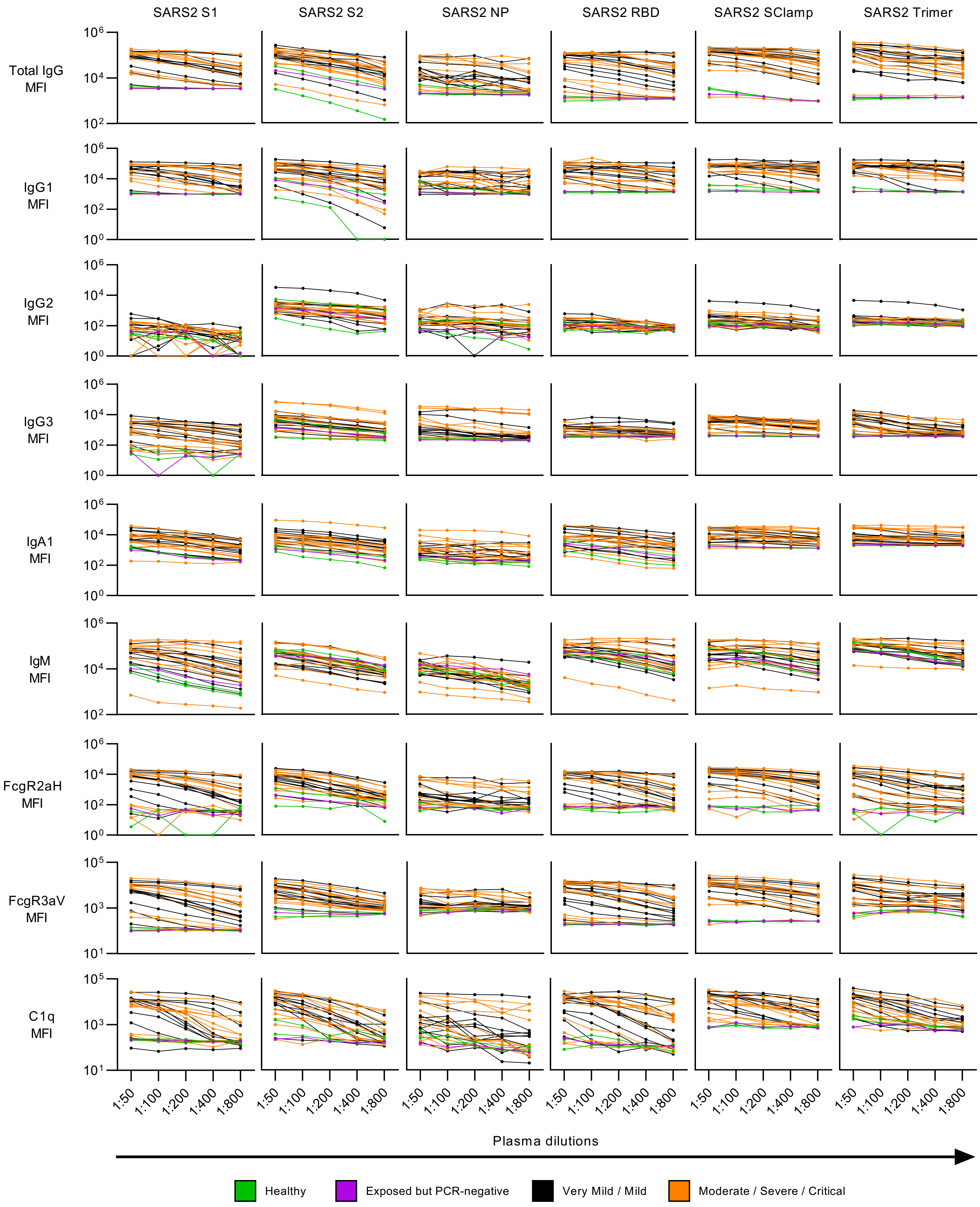

Supplementary Figure 4: SARS2 Ab titrations

Serial dilutions of COVID-19 plasmas against the six SARS-CoV-2 antigens in the assay. Very mild and mild cases were coloured black, while moderate to severe cases were coloured orange. For comparison, two healthy elderly plasma were included (green). D1, who was SARS CoV-2-exposed but remained SARS CoV-2 PCR-negative, was also included (purple).

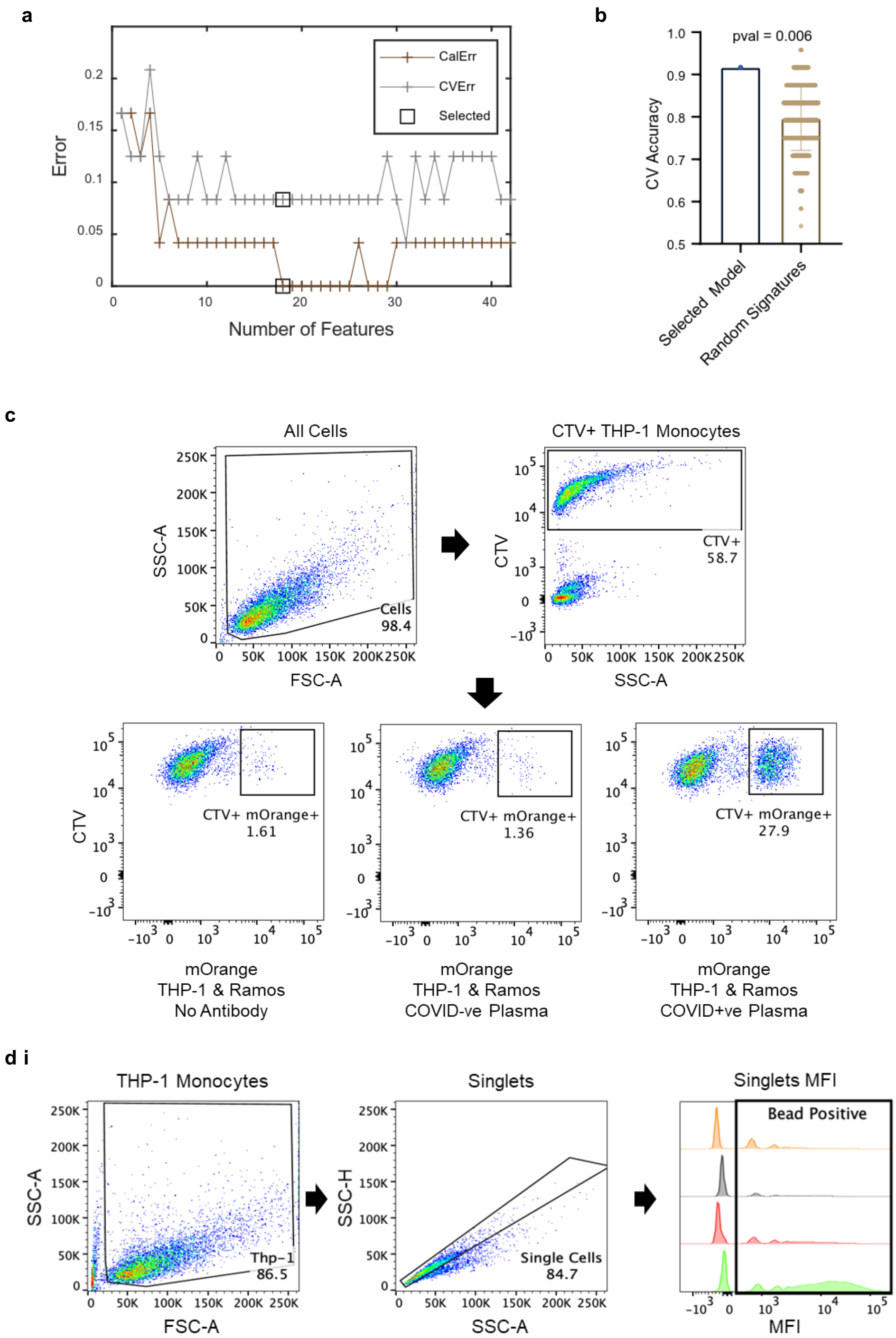

d ii

	Sample ID	Bead Positive Cells		Phagocytosis score (x10^3)
		MFI	%	
	Uncoated beads	1894	47.9	9.07
	S-trimer beads-No Ab	1822	37.1	6.76
	S-trimer beads-COR98	1907	39.4	7.51
	S-trimer beads-CP04 T1	9374	77.4	72.55

Supplementary Figure 5: Feature selection accuracy between COVID+ Children and Elderly model and gating strategy for cell-based assays

An 18-feature model was selected from the 169 Ab features with 100% calibration and 91.37% Cross validation (CV) (a). CV accuracy of selected model compared to performance of n = 500 randomly selected antibody signatures in 500 independent models (p = 0.006, one-sided CV p-value70. Data are presented as mean +/- SD (b). To study association with SARS-CoV-2 S trimer expressing Ramos cells in the presence of Abs, debris-free THP-1 monocytes were first gated based on positive staining with CellTrace TM Violet (CTV), then gated based on their association with Ramos S-orange cells (double positive for CTV and mOrange) (c). The percentage of parent was used to represent the percentage association of Ramos S-orange cells with THP-1 monocytes (% association). To study phagocytosis of SARS-CoV-2 S trimer-coated beads in the presence of Abs, debris-free THP-1 monocyte singlets were first gated for uptake of S-trimer coated or uncoated FITC fluorescent beads (d-i). The geometric mean fluorescent intensity (MFI) and the percentage of FITC bead positive cells (%) were multiplied and then divided by 103 to give the respective phagocytosis score (d-ii). Background subtraction was done using the phagocytosis score of S-trimer beads in the absence of antibody. Cell-based assays were performed in duplicates.
