# Supplementary material for: Systems serology detects functionally distinct coronavirus antibody features in children and elderly
Source: Nat Commun. 2021 Apr 1;12:2037. doi: 10.1038/s41467-021-22236-7 (PMC8016934; doi:10.1038/s41467-021-22236-7)
Supplement: Supplementary file 3 — Description of Additional Supplementary Files. [file 41467_2021_22236_MOESM3_ESM.pdf]

Description of Additional Supplementary Files

Supplementary Data 1: Demographics Healthy Donors

Supplementary Data 2: Concentration of immunoglobulin, Fc and complement factors for healthy donors and COVID-19 patients

Supplementary Data 3: Demographics COVID-19 patients

Supplementary Data 4: Neutralization Data for healthy donors and COVID-19 patients

Supplementary Data 5: Concentration of immunoglobulin, Fc and complement factors for children and elderly COVID-19 patients
