# Supplementary material for: Systems serology detects functionally distinct coronavirus antibody features in children and elderly
Source: Nat Commun. 2021 Apr 1;12:2037. doi: 10.1038/s41467-021-22236-7 (PMC8016934; doi:10.1038/s41467-021-22236-7)
Supplement: Supplementary file 9 — Reporting summary. [file 41467_2021_22236_MOESM9_ESM.pdf]

Corresponding author(s): Katherine Kedzierska & Amy Chung

Last updated by author(s): 28 January 2021

Reporting Summary

Nature Research wishes to improve the reproducibility of the work that we publish. This form provides structure for consistency and transparency in reporting. For further information on Nature Research policies, see our Editorial Policies and the Editorial Policy Checklist.

Statistics

For all statistical analyses, confirm that the following items are present in the figure legend, table legend, main text, or Methods section.

n/a	Confirmed	
☐	☒	The exact sample size (*n*) for each experimental group/condition, given as a discrete number and unit of measurement
☐	☒	A statement on whether measurements were taken from distinct samples or whether the same sample was measured repeatedly
☐	☒	The statistical test(s) used AND whether they are one- or two-sided<br>*Only common tests should be described solely by name; describe more complex techniques in the Methods section.*
☐	☒	A description of all covariates tested
☐	☒	A description of any assumptions or corrections, such as tests of normality and adjustment for multiple comparisons
☐	☒	A full description of the statistical parameters including central tendency (e.g. means) or other basic estimates (e.g. regression coefficient) AND variation (e.g. standard deviation) or associated estimates of uncertainty (e.g. confidence intervals)
☐	☒	For null hypothesis testing, the test statistic (e.g. *F*, *t*, *r*) with confidence intervals, effect sizes, degrees of freedom and *P* value noted<br>*Give P values as exact values whenever suitable.*
☒	☐	For Bayesian analysis, information on the choice of priors and Markov chain Monte Carlo settings
☐	☒	For hierarchical and complex designs, identification of the appropriate level for tests and full reporting of outcomes
☐	☒	Estimates of effect sizes (e.g. Cohen's *d*, Pearson's *r*), indicating how they were calculated

Our web collection on statistics for biologists contains articles on many of the points above.

Software and code

Policy information about availability of computer code

Data collection	FlexMap 3D, Multiskan plate reader, Genpept
Data analysis	Prism-8 (v8.4.1), SPSS Statistics 26, Matlab, Jalview 2.10.5, Ugene 1.16.1

For manuscripts utilizing custom algorithms or software that are central to the research but not yet described in published literature, software must be made available to editors and reviewers. We strongly encourage code deposition in a community repository (e.g. GitHub). See the Nature Research guidelines for submitting code & software for further information.

Data

Policy information about availability of data

All manuscripts must include a data availability statement. This statement should provide the following information, where applicable:

- Accession codes, unique identifiers, or web links for publicly available datasets
- A list of figures that have associated raw data
- A description of any restrictions on data availability

Raw source data will be made available in an accompanying "Source Data.lsx" file

Field-specific reporting

Life sciences study design

All studies must disclose on these points even when the disclosure is negative.

Sample size	The sample size was determined by the availability of samples
Data exclusions	No data were excluded
Replication	Due to the limited availability of the samples, data were not replicated for each individual donor, except for one COVID-19 positive sample (AH0073) which was taken along in every assay as an internal control. Assays for Pan-IgG, IgA1, IgM, C1q, FcgR2aH and FcgR3aV were performed in duplicates.
Randomization	N/A
Blinding	Experiments were not blinded

Reporting for specific materials, systems and methods

We require information from authors about some types of materials, experimental systems and methods used in many studies. Here, indicate whether each material, system or method listed is relevant to your study. If you are not sure if a list item applies to your research, read the appropriate section before selecting a response.

Materials & experimental systems

n/a	Involved in the study	
☐	☒	Antibodies
☐	☒	Eukaryotic cell lines
☒	☐	Palaeontology and archaeology
☒	☐	Animals and other organisms
☐	☒	Human research participants
☒	☐	Clinical data
☒	☐	Dual use research of concern

Methods

n/a	Involved in the study	
☒	☐	ChIP-seq
☐	☒	Flow cytometry
☒	☐	MRI-based neuroimaging

Antibodies

Antibodies used	We used commercially-available antibodies as per Materials and Methods
Validation	Antibodies were validated by the manufacturers and titrated in our laboratory prior to their use

Eukaryotic cell lines

Policy information about cell lines

Cell line source(s)	THP-1 and Ramos cell lines (ATCC)
Authentication	THP-1 were authenticated by testing for cell surface markers CD14, CD16, CD32, CD64, while Ramos cells were used as a target cells by transfection with SARS-CoV2 spike trimer and mOrange and were authenticated by surface staining with SARS-CoV2 RBD specific mAbs and mOrange expression via flow cytometry
Mycoplasma contamination	Original stocks of frozen cell lines were tested for mycoplasma, however passages of both cell lines were not recently tested.
Commonly misidentified lines (See ICLAC register)	*Name any commonly misidentified cell lines used in the study and provide a rationale for their use.*

Human research participants

Policy information about studies involving human research participants

Population characteristics	Please refer to Extended Data Figure 2a for an overview of our Cohort, Extended Data Table 1 for details of the healthy donor cohort, Extended Data Table 3 for details of our COVID-19 patient cohort
Recruitment	Healthy donors <18 yrs of age were recruited at the Launceston General Hospital (Tasmania). Healthy adult donors were recruited via the University of Melbourne (Victoria). Healthy elderly donors were recruited at the Deepdene Medical Clinic (Victoria). SARS-CoV-2-infected patients (age 21-75) were recruited at the Alfred Hospital (AH). Convalescent individuals who recovered from COVID-19 were recruited by University of Melbourne or James Cook University (Queensland). All donors or their legal guardians provided written informed consent prior to participation. SARS-COV2 infected donors (<20) were recruited at the Murdoch Children's Research Institute (Melbourne) or by the University of Melbourne.
Ethics oversight	Human experimental work was conducted according to the Declaration of Helsinki principles and according to the Australian National Health and Medical Research Council Code of Practice. The study was approved by the Human Research Ethics Committee (HREC) of the University of Melbourne (Ethics ID #1443389.4, #2056761, #1647326, 1955465, #2056689) for healthy adult and elderly donors, Tasmanian Health and Medical HREC (H0017479) for healthy child donors, and by Alfred Hospital (#280/14) for COVID-19 patients, James Cook University (#H7886) and University of Melbourne (#2056689) for convalescent individuals. RCH HREC (#63666) for SARS-CoV-2 infected children (<20yrs)

Note that full information on the approval of the study protocol must also be provided in the manuscript.

Flow Cytometry

Plots

Confirm that:

- [x] The axis labels state the marker and fluorochrome used (e.g. CD4-FITC).
- [x] The axis scales are clearly visible. Include numbers along axes only for bottom left plot of group (a 'group' is an analysis of identical markers).
- [x] All plots are contour plots with outliers or pseudocolor plots.
- [x] A numerical value for number of cells or percentage (with statistics) is provided.

Methodology

Sample preparation	THP-1 monocytes (10,000/well) were added to opsonized beads and incubated for 16 hours under cell culture conditions. Cells were fixed with 2% formaldehyde and acquired by flow cytometry
Instrument	BD LSR Fortessa with a HTS
Software	FlowJo 10.7.1
Cell population abundance	THP-1 cell line was used
Gating strategy	Described in Extended Figure 5 (THP-1 monocytes->singlets->Bead positive)

- [x] Tick this box to confirm that a figure exemplifying the gating strategy is provided in the Supplementary Information.
